# Corpus callosum lesions are associated with worse cognitive performance in cerebral amyloid angiopathy

**DOI:** 10.1093/braincomms/fcac105

**Published:** 2022-04-26

**Authors:** Whitney M. Freeze, Maria Clara Zanon Zotin, Ashley A. Scherlek, Valentina Perosa, Corinne A. Auger, Andrew D. Warren, Louise van der Weerd, Dorothee Schoemaker, Mitchell J. Horn, M. Edip Gurol, Elif Gokcal, Brian J. Bacskai, Anand Viswanathan, Steven M. Greenberg, Yael D. Reijmer, Susanne J. van Veluw

**Affiliations:** 1 Department of Radiology, Leiden University Medical Center, Leiden, The Netherlands; 2 Department of Neuropsychology and Psychiatry, Maastricht University, Maastricht, The Netherlands; 3 Department of Neurology, J. Philip Kistler Stroke Research Center, Massachusetts General Hospital, Boston, MA, USA; 4 Department of Medical Imaging, Hematology and Clinical Oncology, Ribeirão Preto Medical School, USP, SP, Brazil; 5 MassGeneral Institute for Neurodegenerative Disease, Massachusetts General Hospital, Charlestown, MA 02129, USA; 6 Department of Psychiatry, Massachusetts General Hospital, Boston, MA, USA; 7 Department of Neurology, Brain Center Rudolf Magnus, University Medical Center Utrecht, Utrecht, The Netherlands

**Keywords:** corpus callosum, cognition, cerebral amyloid angiopathy, diffusion tensor imaging

## Abstract

The impact of vascular lesions on cognition is location dependent. Here, we assessed the contribution of small vessel disease lesions in the corpus callosum to vascular cognitive impairment in cerebral amyloid angiopathy, as a model for cerebral small vessel disease. Sixty-five patients with probable cerebral amyloid angiopathy underwent 3T magnetic resonance imaging, including a diffusion tensor imaging scan, and neuropsychological testing. Microstructural white-matter integrity was quantified by fractional anisotropy and mean diffusivity. *Z*-scores on individual neuropsychological tests were averaged into five cognitive domains: information processing speed, executive functioning, memory, language and visuospatial ability. Corpus callosum lesions were defined as haemorrhagic (microbleeds or larger bleeds) or ischaemic (microinfarcts, larger infarcts and diffuse fluid-attenuated inversion recovery hyperintensities). Associations between corpus callosum lesion presence, microstructural white-matter integrity and cognitive performance were examined with multiple regression models. The prevalence of corpus callosum lesions was confirmed in an independent cohort of memory clinic patients with and without cerebral amyloid angiopathy (*n* = 82). In parallel, we assessed corpus callosum lesions on *ex vivo* magnetic resonance imaging in cerebral amyloid angiopathy patients (*n* = 19) and controls (*n* = 5) and determined associated tissue abnormalities with histopathology. A total number of 21 corpus callosum lesions was found in 19/65 (29%) cerebral amyloid angiopathy patients. Corpus callosum lesion presence was associated with reduced microstructural white-matter integrity within the corpus callosum and in the whole-brain white matter. Patients with corpus callosum lesions performed significantly worse on all cognitive domains except language, compared with those without corpus callosum lesions after correcting for age, sex, education and time between magnetic resonance imaging and neuropsychological assessment. This association was independent of the presence of intracerebral haemorrhage, whole-brain fractional anisotropy and mean diffusivity, and white-matter hyperintensity volume and brain volume for the domains of information processing speed and executive functioning. In the memory clinic patient cohort, corpus callosum lesions were present in 14/54 (26%) patients with probable and 2/8 (25%) patients with possible cerebral amyloid angiopathy, and in 3/20 (15%) patients without cerebral amyloid angiopathy. In the *ex vivo* cohort, corpus callosum lesions were present in 10/19 (53%) patients and 2/5 (40%) controls. On histopathology, ischaemic corpus callosum lesions were associated with tissue loss and demyelination, which extended beyond the lesion core. Together, these data suggest that corpus callosum lesions are a frequent finding in cerebral amyloid angiopathy, and that they independently contribute to cognitive impairment through strategic microstructural disruption of white-matter tracts.

## Teaser

Freeze *et al*. report that corpus callosum lesions are a frequent finding in cerebral amyloid angiopathy, and that they independently contribute to cognitive impairment through strategic microstructural disruption of white-matter tracts.

## Graphical Abstract

**Graphical Abstract:**
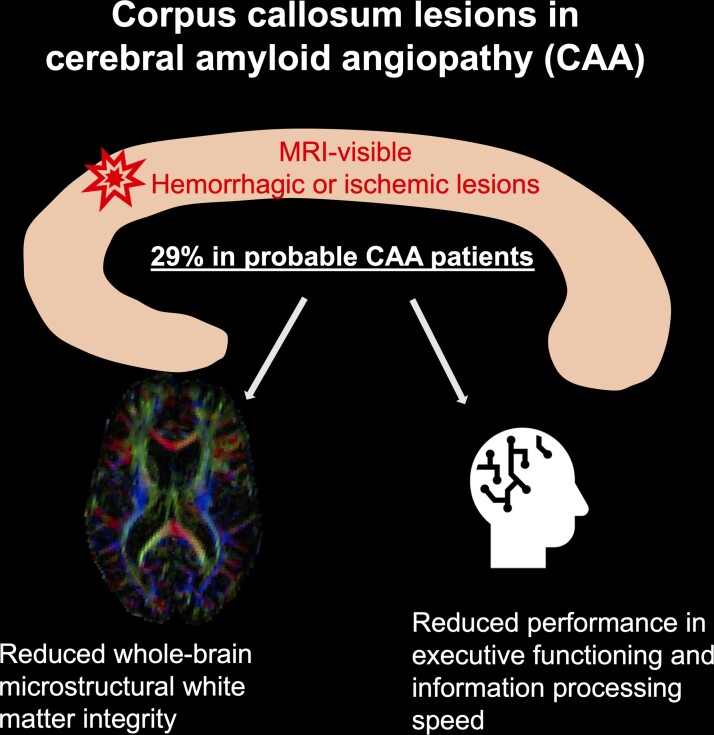
We assessed the prevalence and impact of corpus callosum lesions on whole brain microstructural white matter integrity and cognitive performance in patients with cerebral amyloid angiopathy (CAA). Corpus callosum lesions were prevalent in patients with CAA (29%) and their presence was significantly associated with reduced whole-brain microstructural white matter integrity and reduced cognitive performance in the domains of executive functioning and information processing speed.

## Introduction

Cerebral small vessel disease (cSVD) is a major contributor to vascular cognitive impairment. The impact of vascular lesions on cognition strongly depends on the lesion location, with lesions located in strategic brain regions being more relevant in explaining cognitive impairment than global lesion volume.^1,2^

Cerebral amyloid angiopathy (CAA) is a common type of cSVD and is pathologically characterized by the deposition of amyloid-β within the small cortical and leptomeningeal arteries in the brain.^3^ On MRI, CAA manifests as both haemorrhagic [i.e. lobar cerebral microbleeds (CMBs), cortical superficial siderosis] and ischaemic [i.e. cerebral microinfarcts, white-matter hyperintensities (WMHs) and lobar lacunar infarcts] tissue injury.^4,5^ In addition to these overt MRI lesions, CAA has been associated with white-matter atrophy^6^ and microstructural tissue injury in the white matter, which can be detected with diffusion tensor imaging (DTI).^7^ These microstructural abnormalities are characterized by changes in fractional anisotropy (FA) and mean diffusivity (MD) DTI measures, which quantify the directional dependency and degree of water diffusion within each voxel. A post-mortem MRI study in patients with CAA has demonstrated that DTI changes correspond to tissue rarefaction, in particular axonal loss and myelin loss.^8^ Although the exact mechanisms underlying CAA-related DTI changes have not been specified, multiple small white-matter lesions have been suggested to cumulatively contribute to this type of covert tissue injury,^5,9^ possibly by inducing widespread secondary damage beyond the lesion core.^10–13^ Because white-matter diffusion abnormalities are independently associated with cognitive functioning in CAA,^7^ in particular information processing speed and executive functioning, small strategic white-matter lesions may be of high clinical significance.

Previous work in CAA suggests that the white-matter microstructure is not homogeneously disrupted throughout the brain, but that temporal and posterior brain regions are affected at a greater extent and/or earlier than frontal regions.^14,15^ Because of the predilection of CAA pathology for posterior brain regions, previous studies have dedicated attention to microstructural changes within posterior white-matter tracts.^7,15^ In contrast, the strategically located corpus callosum (CC) has received relatively little attention in CAA, despite its importance as the main fibre tract that connects both cerebral hemispheres. Overt lesions in the CC have been associated with neurological deficits in a number of neurological diseases^16,17^ and reductions in CC white-matter integrity are associated with lower cognitive performance.^18,19^ To the best of our knowledge, the occurrence of CC lesions in patients with CAA has not been assessed before and it remains unknown whether they independently contribute to cognitive impairment. We addressed these outstanding questions in a cohort of patients with probable CAA, recruited through our stroke clinic. In addition, we assessed the prevalence of CC lesions in an independent cohort of memory clinic patients with and without probable CAA to check whether the prevalence of CC lesions is comparable in patients with CAA recruited from a different source and in non-CAA patients with cognitive problems. Finally, we assessed *ex vivo* MRI scans of intact hemispheres in a third cohort of patients with pathologically confirmed CAA and controls to determine the histopathological nature of CC lesions, to check for the presence of CAA in their immediate vicinity and to explore the severity of peri-lesional damage. Studying the CC in CAA, as a model for cSVD in general, will clarify the contribution of CC lesions to vascular cognitive impairment.

## Materials and methods

### Participants stroke clinic cohort

Patients were included through an ongoing single-centre longitudinal cohort study at Massachusetts General Hospital (MGH). Details on patient selection and inclusion have been described previously.^15^ In short, non-demented patients with probable CAA defined by the modified Boston criteria^20,21^ were prospectively recruited through the stroke clinic. Participants with a diagnosis of neurological disease other than CAA or MRI contraindications were excluded from the study. We included all patients with available 3 tesla (T) MRI research scans and a diagnosis of probable CAA according to the modified Boston criteria based on clinical scans at enrolment.^21^ Because the high-resolution research scans revealed additional deep CMBs in a few patients, which were not detected on the clinical scans, we decided the following: Patients with >2 deep CMBs and/or deep intracerebral haemorrhage (ICH) on research scans (*n* = 3) were excluded because it was unclear whether CAA was the predominant type of cSVD in these participants. We decided to keep participants with ≤2 deep CMBs on research scans who otherwise fulfilled the criteria of probable CAA (*n* = 7) as CAA pathology in these patients appeared to be predominant and excluding these patients would lead to a loss of generalizability, and statistical power. It should be noted that these patients fulfilled the criteria for probable CAA based on the clinical scans. However, since a diagnosis of mixed-type cSVD would have been more appropriate based on the research scans, we decided to perform *post hoc* sensitivity analyses by excluding these individuals from the analyses. We used cross-sectional data from the most recent study visit. The study was approved by the MGH Institutional Review Board and informed consent was obtained from all patients or their surrogates.

### *In vivo* MR imaging protocol

The MR images were acquired on a 3T MR imaging system with a 32-channel head coil (Siemens, Magnetom Prisma-Fit). The protocol included a 3D T_1_-weighted multi-echo scan [repetition time (TR), 2510 ms; echo time (TE), 1.69, 3.55, 5.41, 7.27 ms; voxel size, 1.0 mm^3^ isotropic], a 3D T_2_-weighted fluid-attenuated inversion recovery (FLAIR) scan (TR, 5000 ms; TE, 356 ms; inversion time, 1800 ms; voxel size, 0.90 mm^3^ isotropic) a susceptibility-weighted imaging (SWI) scan (TR, 30 ms; TE, 20 ms; voxel size, 0.90 × 0.90 × 1.4 mm^3^), a T_2_*-weighted gradient-echo scan (GRE; TR, 500 ms; voxel size, 2.0 mm^3^ isotropic) and a diffusion-weighted scan (TR, 8000 ms; TE, 82 ms; voxel size, 2.0 mm^3^ isotropic; 64 gradient directions with a *b*-value of 700 s/mm^2^ and one *b* = 0 s/mm^2^ image).

### Diffusion tensor imaging processing

Diffusion-weighted imaging scans were analysed and processed in ExploreDTI (www.exploredti.com). Corrections for subject motion and eddy current-induced geometric distortions were applied.^22^ Affine transformation was used for registration of a normalized white-matter atlas to each individual DTI scan.^23^ The average FA and MD of the CC (not excluding any lesions) and the rest of the white matter (excluding the CC and corticospinal tract because the brainstem was not included in the field of view for all participants) were computed for each participant.^23^ In patients with unilateral ICH (*n* = 31), we excluded the diffusion metrics in the affected hemisphere to account for the possible effects of ICH on the microstructural white-matter integrity.

### Neuroimaging markers

Conventional CAA-related imaging markers were scored by two experienced raters (M.C.Z.Z. and E.G.) who were blinded to clinical and neuropsychological data. Presence or absence of ICH and number of CMBs (excluding CMBs close to ICH), presence or absence of cortical superficial siderosis and the number of cerebral microinfarcts were scored based on established criteria.^13,24,25^

The WMH volume was calculated using an automated method similar to the one previously described.^26,27^ In short, T_1_-weighted images were volumetrically segmented with FreeSurfer (http://surfer.nmr.mgh.harvard.edu/). Subsequently, each patient’s FLAIR image was skull stripped and registered to the reconstructed T_1_-weighted images. The registered FLAIR was then masked and binarized using the tissue segmentation maps to exclude all non-white-matter contributions. Abnormal white matter was identified through a threshold of voxel intensity of mean white matter + 1.3 standard deviation (SD), which was the optimal threshold for this data set. Only clusters of a minimum of 30 contiguous voxels exhibiting hyperintensity beyond the threshold were included in the final calculation. In patients with unilateral ICH (*n* = 31), we excluded the WMH volume in the affected hemisphere, and multiplied the WMH volume in the unaffected hemisphere by two to account for the possible effects of ICH on WMH volume. Normalized WMH was expressed as the percentage of estimated intracranial volume (ICV) from the FreeSurfer parcellation. To calculate normalized brain volume, the BrainSegNotVent variable from the FreeSurfer parcellation was used, and expressed as a percentage of ICV.

The CC lesions were scored by two experienced raters (W.M.F. and S.J.v.V.), who were blinded to clinical and neuropsychological data, on T_1_, FLAIR and blood-sensitive sequences (SWI and/or GRE). CC lesions were classified as ischaemic or haemorrhagic. Presumed ischaemic CC lesions included microinfarcts (focal demarcated lesions typically <5 mm in size),^13^ larger infarcts (including lacunar infarcts characterized by cavitation with a hyperintense rim on FLAIR images)^25^ and diffuse FLAIR hyperintensities, which typically had a bilateral symmetrical shape in the CC. Haemorrhagic CC lesions included CMBs^25^ and ICH extension into the CC. The location of each lesion was noted (genu, body and/or splenium). Inter-rater reliability for the presence of CC lesions was substantial [κ (95% confidence interval, CI) = 0.76 (0.58–0.94)]. Few discrepancies were resolved during a consensus meeting.

### Clinical evaluation

Demographic variables and medical history were recorded. A brief cognitive screening test (Mini-Mental State Examination) and standardized neuropsychological test battery were administered to assess cognitive performance. Composite scores were computed for the following cognitive domains based on neuropsychological theory^28^: executive functioning (Controlled Oral Word Association test—FAS; Trail Making Test B; Digit Span Backward), processing speed and attention (Trail Making Test A; WAIS-III Digit-Symbol Coding; Digit Span Forward), memory (Hopkins Verbal Learning Test, delayed recall; Free and Cued Selective Reminding Test, free recall), language [Boston Naming Test (15 items); Semantic Fluency (animals)] and visuospatial processing [Benton Facial Recognition test (short form); Benton Judgement of Line Orientation (30 items)]. *Z*-scores for each test were calculated based on the mean and SD of the whole study sample and averaged across tests to obtain a single average *Z*-score per cognitive domain.

### Participants memory clinic cohort

The presence of CC lesions was assessed in an independent cohort of memory clinic patients, which included both patients with and without probable CAA (Supplementary Table 1). The study was designed to include around 50% of patients with CAA and 50% of patients with (mild) cognitive impairment without CAA. They underwent the same MR imaging protocol as described for the stroke clinic cohort above. Further details on this cohort have been described elsewhere.^29^ The study was approved by the MGH Institutional Review Board and informed consent was obtained from all patients or their surrogates. For practical reasons, CC lesions in this cohort were assessed by a third experienced rater (M.C.Z.Z.) after establishing substantial inter-rater agreement on a subset of images from the stroke clinic cohort [κ (95% CI) = 0.74 (0.4–1)].

### *Ex vivo* cohort

Intact human brain hemispheres from 19 patients with definite CAA^20^ and 5 non-CAA control cases were included from an ongoing post-mortem MRI study at MGH. Details on the inclusion process, scanning procedures and tissue block sampling have been described previously.^8,30^ In short, the hemispheres were fixed in 10% formalin for several weeks after autopsy. Prior to *ex vivo* 3T MRI, the hemispheres were placed in a plastic bag filled with periodate-lysine-paraformaldehyde and vacuum sealed. The samples were scanned overnight on a 3T MR system (Siemens, Magnetom trioTim syngo) using a 32-channel head coil. The protocol included a T_2_-weighted turbo-spin echo sequence (TR, 1800 ms; TE, 61 ms; voxel size, 500 μm^3^ isotropic) and a gradient-echo fast low-angle shot (FLASH) sequence (TR, 20 ms; voxel size, 500 μm^3^ isotropic). Presumed ischaemic and haemorrhagic CC lesions were identified by the same raters (W.M.F. and S.J.v.V.) who were blinded to CAA severity or other histopathological findings. Inter-rater reliability for the presence of CC lesions was substantial [κ (95% CI) = 0.75 (0.48–1)].

### Histopathology

After scanning, the hemispheres were cut into 10 mm thick coronal brain slabs. Next, four small tissue blocks were systematically sampled from the frontal, temporal, parietal and occipital lobe and embedded in paraffin, after which 6 μm thick sections were cut on a microtome. Brightfield immunohistochemistry against amyloid-β (Aβ; mouse, clone 6F/3D; Agilent Technologies, Santa Clara, CA, USA; 1:200) was performed as previously described.^8^ Cortical and leptomeningeal CAA severity was evaluated by two independent raters (S.J.v.V. and V.P.) on all Aβ-stained sections using a four-point scale (absent, 0; scant Aβ deposition, 1; some circumferential Aβ, 2; widespread circumferential Aβ, 3),^31^ followed by a consensus rating to obtain a final score.^32^ Scores from the four cortical areas were added to form a single cumulative CAA burden score (0–12) for the cortical and leptomeningeal vessels. Similarly, parenchymal Aβ plaque severity was assessed for each tissue block using a four-point scale (absent, 0; mild, 1; moderate, 2; severe, 3) by the same raters (S.J.v.V. and V.P.), followed by a consensus rating to obtain a final score. Scores from the four cortical areas were added to form a single cumulative Aβ plaque burden score (0–12). On haematoxylin and eosin (H&E)-stained sections, arteriolosclerosis severity was assessed in the white matter for each tissue block using a four-point scale (Grades 0–3) by two raters (V.P. and C.A.A.),^33^ followed by a consensus rating to obtain a final score. Scores from the four areas were added to form a single cumulative arteriolosclerosis burden score (0–12). In addition, MRI-guided selection of tissue blocks with representative CC lesions was performed on three patients and 6 μm thick serial sections were cut on a microtome using anatomical landmarks. Sections were stained with standard Luxol fast blue and/or H&E to characterize the MRI-visible lesions microscopically. Also, a Perls Prussian blue stain was performed to assess the presence of iron in haemorrhagic lesions, and immunohistochemistry against Aβ was performed to assess whether CAA was present within the CC.

The post-mortem study was approved by the MGH Institutional Review board and informed consent was obtained from the next of kin prior to autopsy.

### Statistical analyses

Group differences between patients with and without CC lesions were assessed with independent samples T-tests for continuous variables that followed a normal data distribution, and Mann–Whitney U-tests for continuous variables that followed a non-normal data distribution. Pearson χ^2^ or Fisher’s exact tests were applied for categorical variables. We set out to investigate the relationship between CC lesion presence and microstructural white-matter integrity [FA and MD within the CC, and whole-brain white-matter FA and MD (excluding CC)] with multiple regression models, controlling for age, sex and normalized WMH volume. Normalized WMH volume was log transformed to correct for heteroscedasticity of the residuals. In addition, we assessed associations between CC lesion presence as independent variable and performance on each cognitive domain as dependent variable using multiple linear regression analyses, controlling for age, sex, years of education and time between MRI acquisition and neuropsychological assessment. Subsequently, we tested whether the observed associations were independent of potential confounding variables, including presence of ICH, whole-brain white-matter FA and MD (excluding the CC), normalized WMH volume and normalized brain volume, by first adding each of these variables separately and subsequently adding them all together in the regression model. We repeated the analyses after excluding patients with bilateral ICH (*n* = 17), or patients with deep CMBs (*n* = 7), to assess whether this changed the results. Finally, to estimate the relative importance of CC lesions for cognitive performance, we ran regression models including all markers of white-matter integrity and brain volume [normalized WMH volume, normalized brain volume, whole-brain white-matter FA and MD (excluding CC), and CC lesion presence] as dependent variables, and applied a model decomposition method proposed by Lindeman et al.^34^ as implemented in the R package ‘relaimpo’ (version 2.2–3).^35^ False discovery rate corrections were applied to control for multiple comparisons of five cognitive domains.^36^ Inter-rater reliability for determining the presence of CC lesions was computed with Cohen’s Kappa. Mann–Whitney U-tests were used to assess differences in the prevalence of CC lesions in memory clinic patients with and without probable CAA in the independent cohort. A threshold of *α* < 0.05 was used to determine statistical significance and all *P*-values are two-tailed. Analyses were conducted using R statistical software (version 3.6.1.).^37^

### Data availability

Data can be made available by the corresponding author upon reasonable request.

## Results

### Stroke clinic cohort

**Figure 1:**
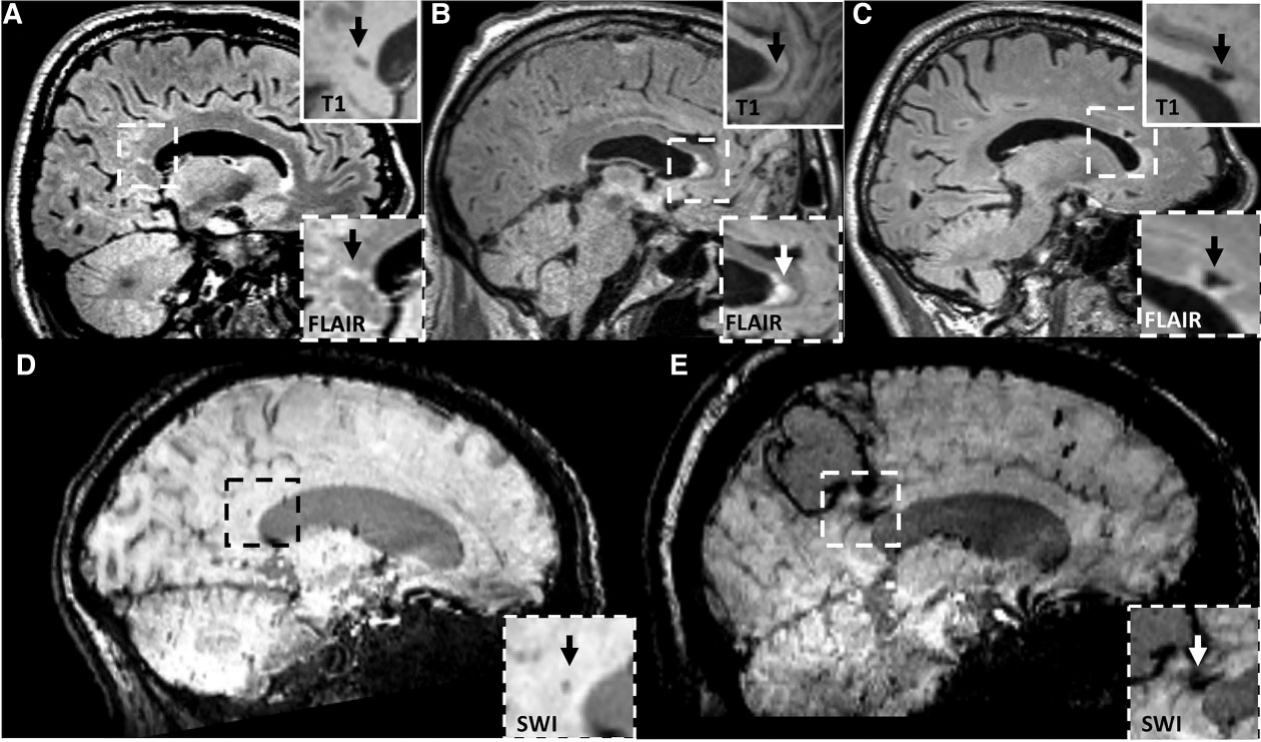
**Representative examples of CC lesions in patients with CAA.** (**A**) Cerebral microinfarct located in the splenium of the corpus callosum. (**B**) Diffuse FLAIR hyperintensity located in the genu of the corpus callosum. (**C**) Lacunar infarct located in the genu of the corpus callosum. (**D**) Cerebral microbleed located in the splenium of the corpus callosum. (**E**) Extension of intracerebral haemorrhage into the splenium of the corpus callosum.

Data from 65 patients with probable CAA were included in this study (see Supplemental Fig. 1 for a flow chart). One patient was excluded from the DTI analyses due to insufficient scan quality. A total number of 21 CC lesions was found in 19 patients (29%): 17 patients had one CC lesion and 2 patients had two CC lesions. Most CC lesions (17/21, 81%) were of ischaemic nature, compared with only a few haemorrhagic lesions (4/21, 19%). The most frequent subtypes were diffuse FLAIR hyperintensities (10/21, 48%), followed by microinfarcts (5/21, 24%), and ICH extension into the CC (3/21, 14%). The remaining three lesions were two larger infarcts and a microbleed. Lesions were most frequently located in the splenium (10/21, 48%), followed by the genu (7/21, 33%), and the body of the CC (2/21, 10%). Two patients had diffuse FLAIR hyperintensities that covered multiple CC regions. Representative examples of each type of CC lesion are shown in Fig. 1. Baseline characteristics of patients with and without CC lesions are described in Table 1. No differences were found for age, sex, years of education, any of the neuroimaging markers or presence of vascular risk factors between patients with and without CC lesions.

**Table 1:** Characteristics of CAA cases with and without CC lesions

	CC lesion absent (*n* = 46)	CC lesion(s) present (*n* = 19)	*P*-value
*Demographics*			
Age, years (SD)	69.0 (8.0)	71.2 (4.0)	0.154
Female, *n* (%)	18 (39)	9 (47)	0.737
Education, years (SD)^a^	16.5 (2.6)	17.0 (4.5)	0.672
*Neuroimaging markers*			
Brain volume (%ICV) (SD)	66.2 (3.8)	63.4 (4.7)	**0**.**027**
ICH present, *n* (%)	34 (74)	14 (74)	1
Lobar CMB, median (range)	31.5 (0–495)	32 (3–740)	0.644
Normalized WMH, median (range)	0.36 (0.00–1.63)	0.30 (0.01–3.20)	0.470
Cortical CMI present, *n* (%)	22 (48)	8 (42)	0.883
≤2 deep CMB present, *n* (%)	13 (6)	5 (1)	0.631
cSS present, *n* (%)	33 (72)	17 (89)	0.223
*Vascular risk factors*			
Hypertension, *n* (%)^a^	18 (42)	10 (53)	0.611
Hypercholesterolemia, *n* (%)^a^	16 (37)	8 (42)	0.935
Type II diabetes mellitus, *n* (%)^a^	3 (7)	1 (5)	1
Cardiac disease, *n* (%)^a^	4 (9)	3 (16)	0.665
Current tobacco use, *n* (%)^a^	0 (0)	1 (5)	0.307
Current alcohol use, *n* (%)^a^	21 (49)	11 (58)	0.702
*Microstructural white-matter integrity*			
CC FA (SD)^a^	0.48 (0.05)	0.42 (0.05)	**<0**.**001**
CC MD (SD)^a^	1.09 × 10^−3^ (9.2 × 10^−4^)	1.18 × 10^−3^ (1.0 × 10^−3^)	**0.** **004**
Total WM FA (SD)^a^	0.38 (0.03)	0.34 (0.04)	**0.** **002**
Total WM MD (SD)^a^	1.06 × 10^−3^ (7.0 × 10^−4^)	1.14 × 10^−3^ (1.1 × 10^−3^)	**0.** **01**
*Cognition*			
MMSE, median (range)^a^	28 (21–30)	28 (21–30)	0.08
Executive functioning (SD)^a^	0.21 (0.68)	−0.56 (0.72)	**<0**.**001**
Processing speed (SD)^a^	0.20 (0.50)	−0.53 (1.0)	**0**.**008**
Memory (SD)^a^	0.17 (0.93)	−0.39 (0.90)	**0**.**032**
Language (SD)^a^	0.10 (0.87)	−0.34 (1.1)	0.130
Visuospatial processing (SD)^a^	0.15 (0.72)	−0.40 (0.99)	**0**.**047**

Values represent mean (SD) unless otherwise specified. Group comparisons were performed using Mann–Whitney U-tests, χ^2^ tests or Fisher’s exact test when applicable. Significant differences (*P* < 0.05) are depicted in bold.

CC, corpus callosum; ICH, intracerebral haemorrhage; CMB, cerebral microbleeds; CMI, cerebral microinfarcts; WMH, white-matter hyperintensities; FA, fractional anisotropy; MD, mean diffusivity; cSS, cortical superficial siderosis.

^a^ Data were missing: education, *n* = 2; hypertension, *n* = 3; hypercholesterolemia, *n* = 3; type II diabetes mellitus, *n* = 3; cardiac disease, *n* = 3; current tobacco use, *n* = 3; current alcohol use, *n* = 3; CC FA, *n* = 1; CC MD, *n* = 1; total WM FA, *n* = 1; total WM MD, *n* = 1; MMSE, *n* = 1; executive functioning, *n* = 1; information processing speed, *n* = 1; memory, *n* = 2; language, *n* = 1; visuospatial processing, *n* = 3.

### CC lesions and microstructural white-matter integrity

After correcting for age, sex and normalized WMH, CC lesion presence was significantly associated with decreased microstructural white-matter integrity within the CC [standardized beta coefficient (95% CI) FA = −0.467 (−0.685, −0.248), *P* < 0.001; MD = 0.343 (0.115–0.572), *P* = 0.005] and in the whole-brain white matter [excluding the CC; standardized beta coefficient (95% CI) FA = −0.414 (−0.634, −0.194), *P* < 0.001; MD = 0.337 (0.111–0.562), *P* = 0.005]. The results did not change when patients with bilateral ICH or patients with deep CMBs were excluded from the analyses.

### CC lesions and cognition

**Figure 2:**
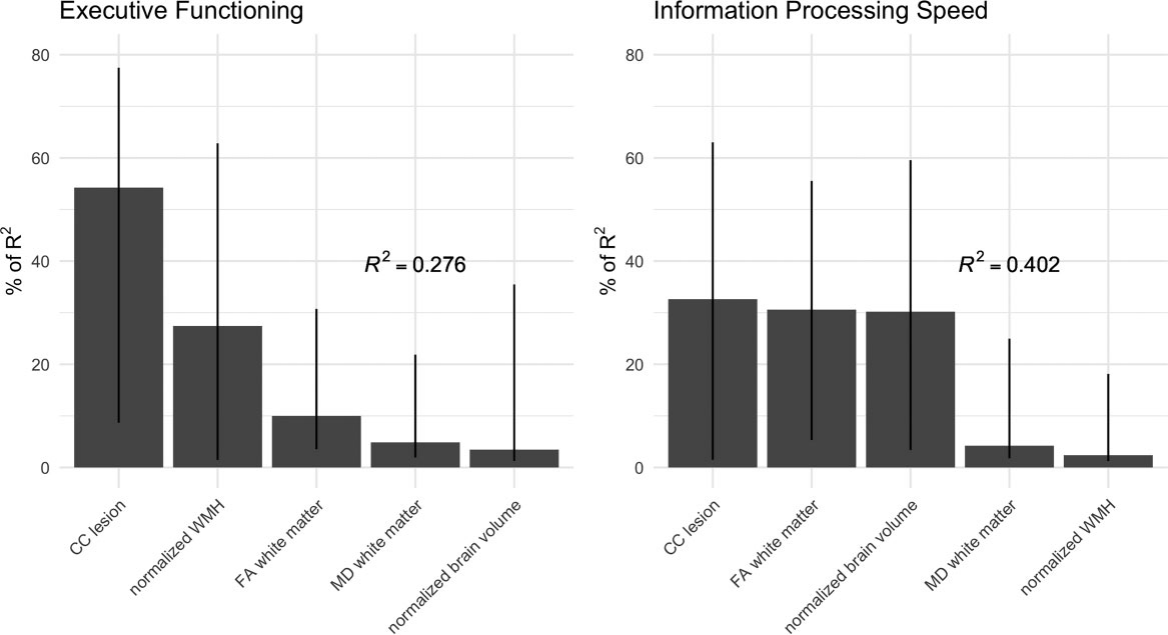
**Association between imaging markers of white-matter integrity and cognitive performance.** The contribution of each imaging marker [CC lesion presence, normalized WMH volume, normalized brain volume, FA in the whole white matter (except CC) and MD in the whole white matter (except CC)] to the total explained variance in multiple regression models with information processing speed (*n* = 63) and executive functioning (*n* = 63) as outcome variable as estimated by the Lindeman *et al*.^34^ method. Lines represent 95% confidence intervals after bootstrapping.

After correcting for age, sex, years of education and time between MRI and neuropsychological assessment, CC lesion presence was significantly associated with decreased performance on the domains of executive functioning, processing speed, memory and visuospatial functioning (Table 2). After correction for additional confounding variables, including presence of ICH, microstructural white-matter integrity (FA and MD in the whole-brain white matter), and normalized WMH and brain volume, CC lesion presence was significantly associated with executive functioning and processing speed, but no longer with memory and visuospatial functioning. The results did not change when patients with bilateral ICH or deep CMBs were excluded from the analyses (Table 2). In regression models including CC lesion presence, normalized WMH volume, normalized brain volume and whole-brain FA and MD as independent variables, and executive functioning and processing speed as dependent variables, the total explained variance (*R*^2^) was 27.6 and 40.2% (Fig. 2). CC lesion presence explained around 55% (executive functioning) and 33% (information processing speed) of this total explained variance in cognitive performance, so around 15 and 13% of the total explained variance (Fig. 2).

**Table 2:** Associations between CC lesions and cognition

Standard covariates	Additional covariate (potential confounders)					All covariates together
Age, sex, education, time between NPA and MRI	ICH presence	Whole brain FA (except CC)	Whole brain MD (except CC)	Normalized WMH	Normalized brain volume	
*Executive functioning*						
**-0.726 (−1.118, −0.334),** ***P* < 0.001**	**−0.722 (−1.110, −0.335),** ***P* < 0.001**	**−0.680 (−1.122, −0.238),** ***P* = 0.003**	**−0.714 (−1.139, −0.290),** ***P* = 0.001**	**−0.645 (−1.028, −0.262),** ***P* = 0.001**	**−0.452 (−1.174, −0.345),** ***P* = 0.001**	**−0.427 (−1.168, −0.255),** ***P* = 0.003**
*Information processing speed*						
**−0.709 (−1.108, −0.309),** ***P* < 0.001**	**−0.707 (−1.108, −0.306),** ***P* < 0.001**	**−0.728 (−1.182, −0.274),** ***P* = 0.002**	**−0.750 (−1.185, −0.315),** ***P* = 0.001**	**−0.602 (−0.980, −0.225),** ***P* = 0.002**	**−0.322 (−0.940, −0.144),** ***P* = 0.009**	**−0.312 (−0.980, −0.122),** ***P* = 0.013**
*Memory*						
**−0.593 (−1.098, −0.087),** ***P* = 0.022**	**−0.590 (−1.098, −0.082),** ***P* = 0.024**	**−0.670 (−1.126, −0.144),** ***P* = 0.015**	**−0.653 (−1.189, 0.118),** ***P* = 0.018**	**−**0.512 (**−**1.017, **−**0.006), *P* = 0.047	**−**0.232 (**−**0.994, 0.059), *P* = 0.081	**−**0.294 (**−**1.152, **−**0.013), *P* = 0.045
*Language*						
**−**0.345 (**−**0.853, 0.114), *P* = 0.180	**−**0.342 (**−**0.851, 0.167), *P* = 0.184	**−**0.369 (**−**0.939, 0.202), *P* = 0.201	**−**0.404 (**−**0.9248 0.140), *P* = 0.143	**−**0.225 (**−**0.716, 0.265), *P* = 0.361	**−**0.161 (**−**0.874, 0.205), *P* = 0.219	**−**0.138 (**−**0.849, 0.281), *P* = 0.318
*Visuospatial processing*						
**−0.552 (−0.980, −0.124),** ***P* = 0.013**	**−0.555 (−0.986, −0.123),** ***P* = 0.013**	**−0.590 (−1.073, 0.109),** ***P* = 0.017**	**−0.638 (−1.096, −0.180),** ***P* = 0.007**	**−0.544 (−0.978, −0.109),** ***P* = 0.015**	**−**0.244 (**−**0.881, **−**0.013), *P* = 0.044	**−**0.261 (**−**0.970, 0.012), *P* = 0.056

Values represent beta coefficients (95% confidence interval) of CC lesion presence. Significant associations after false discovery rate correction are depicted in bold.

CC, corpus callosum; FA, fractional anisotropy; MD, mean diffusivity; NPA, neuropsychological assessment.

### Memory clinic patient cohort

A total number of 82 memory clinic patients were included in this study, of which 54 fulfilled criteria for probable CAA, 8 possible CAA and 20 were considered non-CAA control patients [this group included both patients with (*n* = 8) and without (*n* = 12) MRI markers of cSVD]. CC lesions were present in 14/54 (26%) probable patients with CAA, 2/8 (25%) possible patients with CAA and in 3/20 (15%) memory clinic patients without CAA. We did not find group differences for the presence of CC lesions (Fisher’s exact test *P*-value = 0.608).

### *Ex vivo* cohort

**Figure 3:**
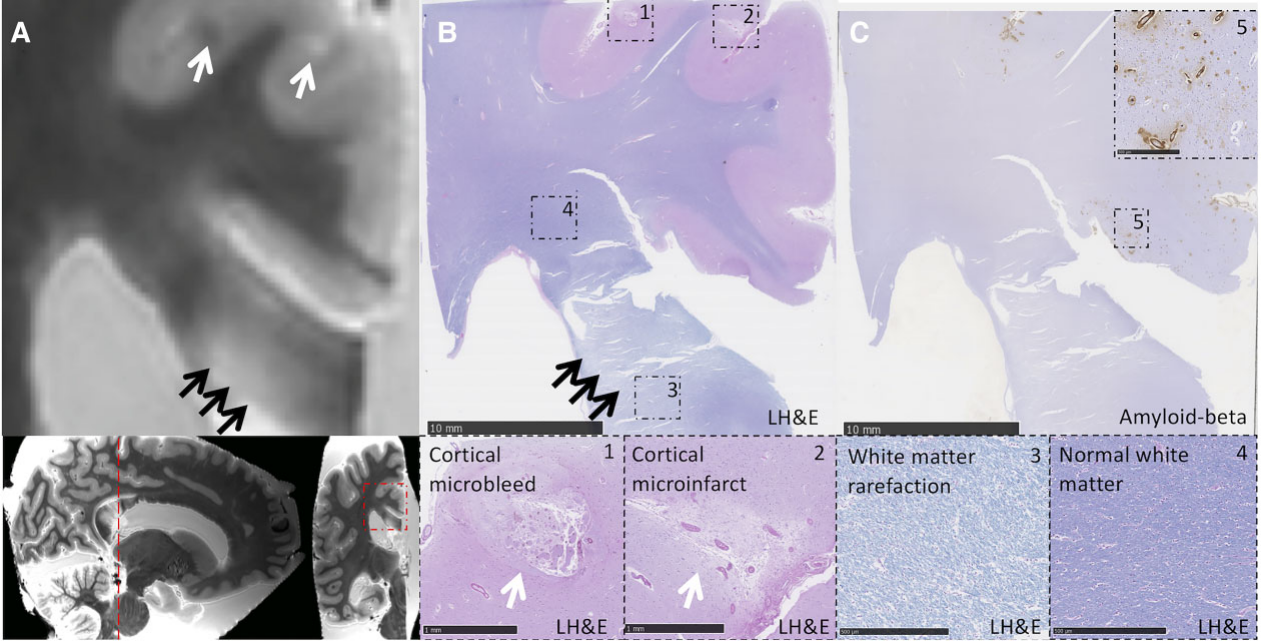
**Diffuse T_2_-hyperintense lesion in the splenium of the corpus callosum.** (**A**) A diffuse T_2_-hyperintense lesion can be observed on coronal T_2_-weighted *ex vivo* MRI (black arrows) in a patient with pathologically confirmed CAA (Case 2). A cortical microbleed (left) and cortical microinfarct (right) can be observed in the adjacent cortex (white arrows). (**B**) The corresponding Luxol fast blue and haematoxylin and eosin (LH&E)-stained section is shown. Insets 1 and 2 show the location of the cortical microbleed (Inset 1) and cortical microinfarct (Inset 2) which were observed on MRI. Inset 3 shows white-matter rarefaction at the location corresponding to the T_2_-hyperintense CC lesion on MRI. Distal from the lesion, the white matter appears normal (Inset 4). (**C**) The presence of CAA pathology was confirmed within the cortex on the adjacent Aβ-stained section (Inset 5). No CAA was observed within the CC.

**Figure 4:**
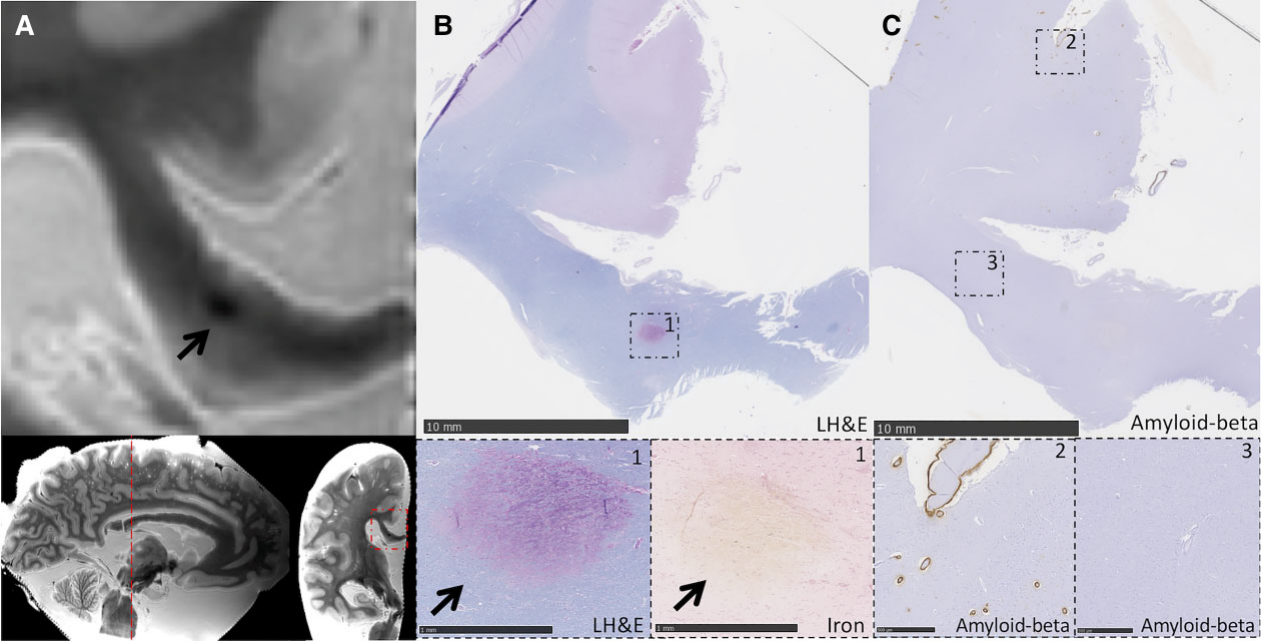
**Cerebral microbleed located in the splenium of the corpus callosum.** (**A**) A CMB located in the splenium of the CC can be observed on coronal T_2_-weighted *ex vivo* MRI (black arrow) in a patient with pathologically confirmed CAA (Case 8). Note that this patient also had participated in the *in vivo* stroke clinic cohort during life. No lesions were observed on the *in vivo* scans that were performed in the same individual approximately 6 months before death (not shown). (**B**) The corresponding Luxol fast blue and haematoxylin and eosin (LH&E)-stained section is shown. The inset shows the presence of lysed red blood cells, indicative of a (sub)acute microbleed (bottom left image, arrow). The relatively acute stage of the microbleed is confirmed by the absence of iron positivity in the adjacent Perls Prussian blue-stained section (bottom right image, arrow). (**C**) The adjacent section stained for Aβ revealed the presence of CAA in the cortex (Inset 2), while vascular Aβ is absent within the corpus callosum (Inset 3).

**Figure 5:**
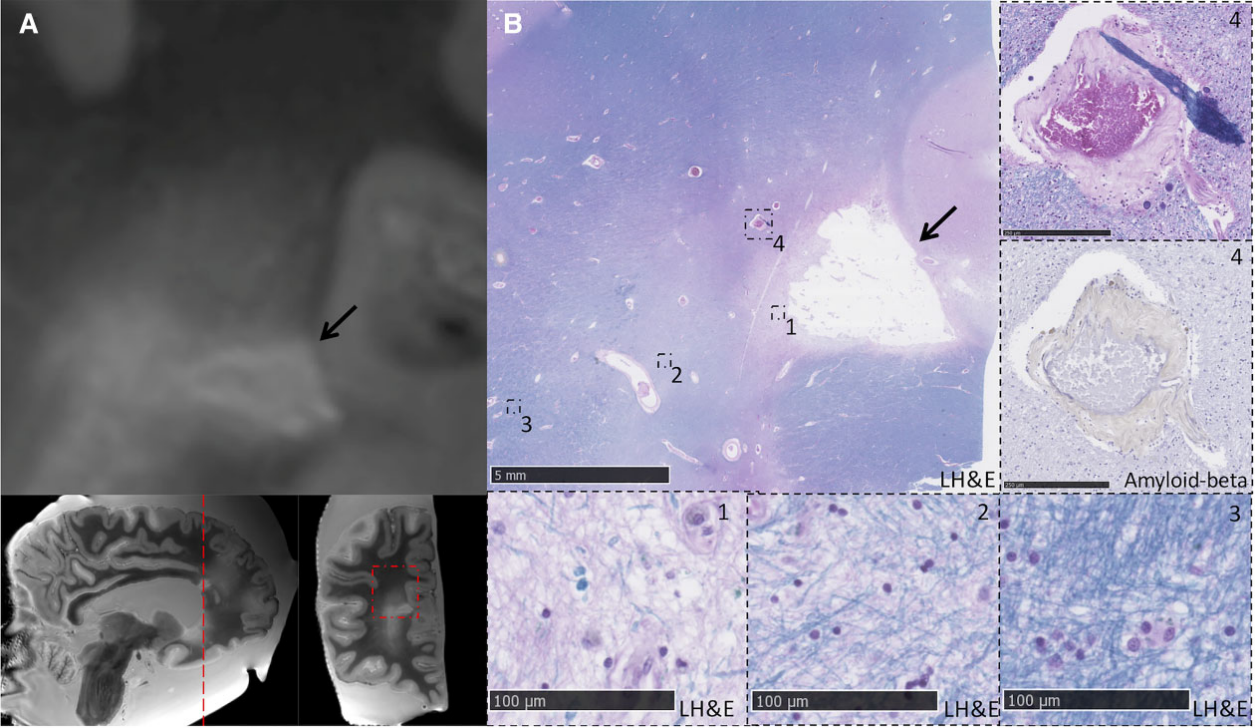
**Lacunar infarct located in the genu of the corpus callosum.** (**A**) A lacunar infarct can be observed in the genu of the CC on T_2_-weighted *ex vivo* MRI (black arrow) in a patient with pathologically confirmed CAA (Case 3). (**B**) The corresponding Luxol fast blue and haematoxylin and eosin (LH&E)-stained section is shown. The cavitated lacunar infarct is indicated by the black arrow. The Insets 1–3 reveal damage to the myelinated fibres in the immediate area surrounding the infarct (Inset 1), as well as in the vicinity of the lesion (Inset 2). At a larger distance, the white matter appears normal (Inset 3). Inset 4 shows an abnormal blood vessel in the vicinity of the lacune. The lipohyalinotic and thickened appearance of the vessel wall, which is negative for Aβ, is suggestive of arteriolosclerosis.

Details of the *ex vivo* cohort are described in Table 3. CC lesions were present in 10/19 patients with CAA (53%) and 2/5 non-CAA controls (40%, Fisher’s exact test *P*-value = 1). Like the *in vivo* stroke clinic cohort, most CC lesions in the patients with CAA (7/10, 70%) were of ischaemic appearance, whereas fewer haemorrhagic lesions were observed (3/10, 30%). Similarly, the most common subtype was diffuse T_2_-hyperintensities (4/10, 40%), followed by microinfarcts (2/10, 20%) and extension of ICH into the CC (2/10, 20%). Only one lacunar infarct (1/10, 10%) and one microbleed (1/10, 10%) were observed. Lesions were located in the splenium (3/10, 30%), genu (3/10, 30%) or body of the CC (1/10, 10%). Three patients with CAA had diffuse T_2_-hyperintensities or ICH extension into the CC that covered multiple CC regions. The CC lesions in the control cases were a diffuse T_2_-hyperintensity and a lacunar infarct, both located in the genu. There were no statistically significant differences in arteriolosclerosis score, Aβ plaque burden and cortical or leptomeningeal CAA scores between patients with CAA with and without CC lesions (all *P* > 0.05). Examples of representative CC lesions on *ex vivo* MRI and corresponding histological findings are shown in Figs 3–5. White-matter rarefaction, axonal damage and demyelination were observed beyond the core of ischaemic lesions (T_2_-hyperintensity and lacunar infarct) (Figs 3–5), but not the microbleed (Fig. 4). Although CAA was prominent in adjacent cortical areas, the walls of blood vessels within the CC were negative for Aβ immunoreactivity.

**Table 3:** Case characteristics *ex vivo* cohort

Case no.	Path diagnosis	Age at death (years)	Sex	Post-mortem interval (h)	Cortical CAA burden score	Leptomeningeal CAA burden score	Amyloid-beta plaque score	Arteriolosclerosis	CC lesion type	CC lesion location
1	CAA	80	M	N/A	5	6	9	8	WMH	Splenium
2	CAA	70	M	16	9	5	9	12	WMH	Multiple
3	CAA	76	M	27	7	8	10	9	Lacune	Genu
4	CAA	65	M	14	7	7	3	7	ICH	Midbody
5	CAA	81	M	N/A	5	7	12	8	WMH	Multiple
6	CAA	70	F	N/A	6	11	9	5	None	
7	CAA	67	M	N/A	10	8	12	11	None	
8	CAA	69	M	36	10	11	7	10	CMB	Splenium
9	CAA	64	F	30	8	11	11	6	WMH	Splenium
10	CAA	79	F	37	8	11	11	8	CMI	Genu
11	CAA	67	M	24	5	12	12	9	None	
12	CAA	88	F	11	8	9	7	5	None	
13	CAA	67	F	16	10	11	12	9	None	
14	CAA	84	F	32	8	9	11	10	None	
15	CAA	67	M	N/A	12	12	8	8	ICH	Multiple
16	CAA	76	M	20	11	12	9	8	None	
17	CAA	78	F	24	7	11	11	8	None	
18	CAA	86	M	20	10	12	10	9	CMI	Genu
19	CAA	85	M	N/A	12	12	11	6	None	
1	CTRL	90	M	6	0	1	9	4	None	
2	CTRL	95	F	4	0	2	1	6	WMH	Genu
3	CTRL	88	F	9	0	1	1	0	None	
4^a^	CTRL	85	M	38	1	4	10	8	CMI	Genu
5	CTRL	82	F	N/A	1	2	2	3	None	

CTRL, control.

^a^ Note that this case had multiple lobar microbleeds during life, but at autopsy only mild CAA was observed in the leptomeningeal vessels and as such considered a CTRL in this study.

## Discussion

In the current study, we reported the prevalence, characteristics and neuroradiological and clinical correlates of CC lesions in patients with CAA. Our main findings can be summarized as follows: (i) CC lesions were prevalent in patients with probable (*in vivo* stroke clinic cohort, 29%; *in vivo* memory clinic patient cohort, 26%) and definite (*ex vivo* cohort, 53%) CAA, and were mostly of ischaemic nature, (ii) patients with CAA with CC lesions had reduced white-matter integrity within the CC and in the rest of the white matter compared with those without CC lesions, (iii) CC lesions were associated with reduced cognitive performance in the domains of information processing speed, executive functioning, memory and visuospatial processing, and (iv) in the domains of information processing speed and executive functioning, this association was independent of other conventional neuroimaging markers of CAA, including ICH presence, WMH volume, brain volume and whole-brain microstructural white-matter integrity. Furthermore, we confirmed the ischaemic and haemorrhagic nature of CC lesions with *ex vivo* MRI and histology, and observed damage to the myelinated white matter within and surrounding ischaemic CC lesions. As expected, CAA pathology was not present within the CC white matter in patients with CAA.

While lesions in the CC are typically associated with neurological diseases such as multiple sclerosis (hyperintense T_2_-lesions have been reported in up to 90% of MS patients)^38,39^ or primary brain tumors,^40,41^ their presence has been previously reported in patients with genetic or rare forms of cSVD. These include cerebral autosomal dominant arteriopathy with subcortical infarcts and leucoencephalopathy (with WMHs as the typical CC lesion type), Binswanger’s disease and Susac syndrome (with small infarcts as the typical lesion type), although the prevalence of CC lesions in these cSVD patient groups remains unclear.^16,41,42^ To our knowledge, the presence of CC lesions has never been assessed before in patients with CAA. This study demonstrates that CC lesions are common MRI findings in CAA and that they are mostly of ischaemic nature (∼70–80%). The prevalence of CC lesions in probable CAA was comparable in both *in vivo* cohorts (29 versus 26%). The higher prevalence in our *ex vivo* cohort (53%) can potentially be attributed to increased disease severity or longer disease duration in patients that came to autopsy, or the higher resolution of the *ex vivo* imaging protocol.

The exact mechanisms underlying the formation of CC lesions in CAA remain unknown. While approximately half of the CC lesions were located in the splenium, which matches the typical posterior predilection for CAA pathology, no CAA-positive blood vessels were found locally in the CC on histology. In a previous study on rare focal CC infarcts, the majority of lesions were also located within the splenium of the CC,^43^ suggesting that the splenium may in general be more vulnerable to ischaemic lesions compared with the genu and midbody. The CC receives blood supply from three arterial systems. The anterior communicating artery branches into the subcallosal and medial callosal arteries which provide the main supply for the anterior part of the CC. The pericallosal branch of the anterior cerebral artery supplies the body of the CC, and the posterior pericallosal artery, a branch from the posterior cerebral artery, supplies the splenium.^44^ Because the CC receives such rich blood supply, large CC infarcts are uncommon,^45^ which was in line with our findings. In contrast, our results suggest that the CC is not equally resilient to focal lesions in the form of small infarcts and haemorrhages. Arteriolosclerosis could be a potential mechanism underlying the formation of small CC lesions, although this study did not reveal an association between hypertension and CC lesion presence.

The presence of CC lesions was associated with reduced microstructural white-matter integrity, not only within the CC itself but also within the rest of the white matter. This association was independent of age and WMH volume. Surprisingly, we did not observe significant associations between CC lesion presence and vascular risk factors or conventional neuroimaging markers of CAA, including the presence of ICH, the presence of cortical superficial siderosis, WMH volume or the number of cortical microinfarcts or lobar microbleeds. Furthermore, we did not observe a significant association between CC lesion presence and cortical or leptomeningeal CAA severity in our *ex vivo* cohort. Together, these findings suggest that CC lesions are not a specific marker for CAA, but rather that (i) CC lesions may independently affect the remote white-matter microstructure, and/or (ii) that CC lesions arise as a consequence of remote and invisible or strategically located neurodegenerative and/or vascular brain damage (i.e. as a secondary mechanism). The first hypothesis is supported by many previous studies that have shown the potential damaging remote effects of focal lesions located in the white matter,^10–12,46,47^ and may apply to focal small infarcts and/or microbleeds within the CC. The second hypothesis may apply to more diffuse FLAIR hyperintense CC lesions and is supported by recent work in animals, showing that Wallerian degeneration following distal strategic cerebral infarction can affect the non-ischaemic CC.^48^ Interestingly, another recent animal study has demonstrated that even a single cortical microinfarct can induce remote damage extending into the contralateral CC.^49^ Moreover, previous work in patients with Alzheimer’s disease and mild cognitive impairment has shown that CC FLAIR hyperintensities are more frequently observed in patients with cognitive impairment compared with cognitively normal controls,^50^ which suggests that neurodegenerative processes (i.e. cortical atrophy) could potentially contribute to the formation of WMH abnormalities within the CC through Wallerian degeneration. Since CAA typically has a posterior predilection, the relatively high prevalence of CC lesions in the splenium could be a secondary consequence of CAA-related haemorrhagic and neurodegenerative posterior cortical damage. Future work could explore these hypotheses further by performing tract-based DTI of the CC and connected cortical brain regions.^8,9^ Such studies should also consider the impact of ischaemic versus haemorrhagic lesion types on the integrity of white-matter fibre bundles, since our *ex vivo* findings suggest that the white-matter integrity is more severely affected around ischaemic compared with haemorrhagic lesions. Furthermore, the prevalence of CC lesions should be explored in cohorts with other types of cSVD and non-cSVD cognitively normal older controls.

The clinical relevance of CC lesions is underlined by their strong association with cognitive performance in multiple cognitive domains. Patients with CC lesions performed on average between 0.5 and 0.9 SDs lower in each cognitive domain compared with patients without CC lesions. The association between CC lesion presence and performance on the domains of memory and visuospatial processing dissipated after correcting for normalized brain volume, which might be explained by the impact of AD- or CAA-related neurodegeneration on these cognitive domains. However, the association between CC lesions and information processing speed and executive functioning was independent of all other neuroimaging markers that were considered relevant in our model. After controlling for the impact of other neuroimaging markers of white-matter integrity and brain volume, the total amount of unique explained variance by CC lesion presence was around 13% for processing speed and around 15% for executive functioning (based on Fig. 2). CC lesions may directly impact cognition by strategically disrupting white-matter connectivity, thereby hampering communication between interhemispheric brain regions. The important association between CC microstructural white-matter integrity and cognitive performance has been previously established in many studies, for example in patients with cSVD,^51^ Parkinson’s disease^19^ or traumatic brain injury.^52^ Furthermore, the CC was identified as an important strategic region for global post-stroke cognitive impairment in a lesion-symptom mapping study.^1^ Our findings now contribute to the existing literature by underlining the clinical relevance of underexplored non-lobar brain damage distal to CAA-affected vessel segments in CAA,^5,8^ which may serve as a model for other sporadic cSVDs. The strong association with processing speed and executive functioning confirms the important role of interhemispheric communication in these cognitive domains.^53,54^

Strengths of this study include the well-characterized cohort of stroke clinic patients with probable CAA, the inclusion of an independent cohort for the assessment of CC lesion prevalence in memory clinic patients with and without probable CAA, and the *ex vivo* validation of CC lesion presence in patients with pathologically confirmed (i.e. definite) CAA. Our results show that CC lesions are a frequent finding in CAA. A limitation is the lack of an age-matched control group to determine whether CC lesions are more frequently observed in patients with CAA compared with healthy individuals. Of note, CC lesions were also observed in 3/20 (15%) of the non-CAA memory clinic patients and 2/5 (40%) of the autopsy non-CAA control cases, which suggests that damage to the CC is likely not specific for CAA, which is in line with previous studies.^16,39^ Please note that we did not hypothesize that CC lesions are specific to CAA, but we aimed to assess their clinical relevance in the context of vascular cognitive impairment. Our findings warrant replication in other CAA and non-CAA cSVD cohorts, recruited from different sources. It would also be interesting to assess whether similar associations with cognitive performance can be found in other neurological conditions in which CC lesions are prevalent, such as demyelinating lesions in MS. Furthermore, we did not have enough statistical power to explore the impact of different lesion types (i.e. haemorrhagic versus ischaemic or focal versus diffuse) on microstructural white-matter integrity and cognition, and for the same reason, we were not able to assess the impact of lesion size or to explore the preferential localization of each lesion type within the CC. Although we controlled for the presence of ICH, and we replicated our findings while excluding cases with bilateral ICH, we did not explore differences in the precise location and volume of the ICH or other distal lesions between cases with and without CC lesions, a topic for future studies. The cause of death in the *ex vivo* cohort, which could have influenced the presence of CC lesions in non-CAA controls, was not known for all individuals. The cross-sectional nature of the present study hampers our ability to draw causative conclusions. Future studies are needed to elucidate the mechanisms underlying CC lesion formation in CAA. Because of their apparent clinical significance, CC lesions deserve more attention in both clinical and research settings.

## Supplementary Material

fcac105_Supplementary_Data

## Abbreviations

Aβ =: amyloid-beta
CAA =: cerebral amyloid angiopathy
CC =: corpus callosum
CMBs =: cerebral microbleeds
cSVD =: cerebral small vessel disease
DTI =: diffusion tensor imaging
FA =: fractional anisotropy
FLAIR =: fluid-attenuated inversion recovery
FLASH =: fast low-angle shot
GRE =: gradient echo
ICH =: intracerebral haemorrhage
MD =: mean diffusivity
MGH =: Massachusetts General Hospital
SWI =: susceptibility-weighted imaging
TE =: echo time
TR =: repetition time
WMHs =: white-matter hyperintensities
