# Supplementary material for: Corpus callosum lesions are associated with worse cognitive performance in cerebral amyloid angiopathy
Source: Brain Commun. 2022 Apr 26;4(3):fcac105. doi: 10.1093/braincomms/fcac105 (PMC9123849; doi:10.1093/braincomms/fcac105)
Supplement: fcac105_Supplementary_Data [file fcac105_supplementary_data.zip › supplemental figure 1.pdf]

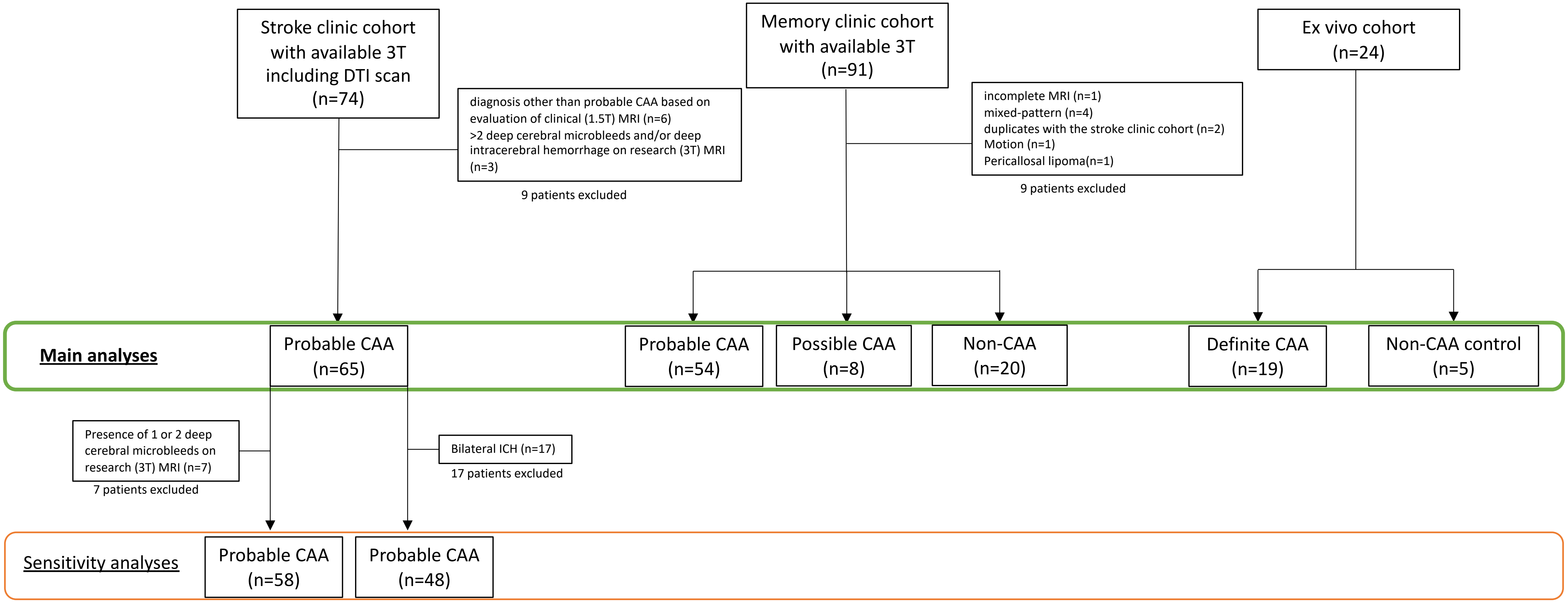

Supplemental figure 1: flow chart of different cohorts that were included. CAA diagnosis was determined based on 1.5T clinical scans for the main analyses. Post-hoc, the analyses were repeated for patients with probable CAA based on the 3T research MRI scans. Furthermore, we repeated the DTI analyses while excluding patients with bilateral ICH to check whether this changed the results.
