# Supplementary material for: Corpus callosum lesions are associated with worse cognitive performance in cerebral amyloid angiopathy
Source: Brain Commun. 2022 Apr 26;4(3):fcac105. doi: 10.1093/braincomms/fcac105 (PMC9123849; doi:10.1093/braincomms/fcac105)
Supplement: fcac105_Supplementary_Data [file fcac105_supplementary_data.zip › Supplementary table 1.docx]

**Supplementary table 1: Basic characteristics of the in vivo validation cohort**

|  | **Total**  *n*=82 | **Probable CAA**  *n*=54 | **Possible CAA**  *n*=8 | **Non-CAA**  *n*=20 | *p*-value |
| --- | --- | --- | --- | --- | --- |
| **Demographics** | | | | | |
| Age (y) (sd) | 73.79 (7.5) | 73.67 (7.84) | 79.41 (6.56) | 71.88 (6.0) | 0.054^a^ |
| Female, n (%) | 37 (45.1) | 23 (42.6) | 4 (50) | 10 (50) | 0.786^b^ |
| **Cognition** | | | | | |
| MMSE, median[range]#  Dementia, n(%)# | 26 (24-28)  22 (27.8) | 26 (24-28)  13 (25.5) | 24.5 (22.25-27.25)  4 (50) | 26.5 (24.25-29)  5 (25) | 0.207^c^  0.373^b^ |
| **Neuroimaging** | | | | | |
| Presence CC lesions, n (%) | 19 (23.2) | 14 (25.9) | 2 (25) | 3 (15) | 0.608^b^ |

# Missing cases: 3 from Probable-CAA

^a^ one-way ANOVA

^b^ Fisher exact test

^c^ Kruskall Wallis test

A threshold of α<0.05 was used to determine statistical significance and all *p*-values are two-tailed
